# LAMP-1 Chimeric to HIV-1 p55Gag in the Immunization of Neonate Mice Induces an Early Germinal Center Formation and AID Expression

**DOI:** 10.3390/vaccines10081246

**Published:** 2022-08-03

**Authors:** Franciane Mouradian Emidio Teixeira, Luana de Mendonça Oliveira, Anna Julia Pietrobon, Érika Machado de Salles, Maria Regina D’Império Lima, Isabelle Freire Tabosa Viana, Roberto Dias Lins, Paula Ordonhez Rigato, Ernesto Torres de Azevedo Marques, Alberto José da Silva Duarte, Maria Notomi Sato

**Affiliations:** 1 Laboratory of Dermatology and Immunodeficiencies, LIM-56, Department of Dermatology, Tropical Medicine Institute of São Paulo, University of São Paulo Medical School, São Paulo 05403000, Brazil; franciane.mteixeira@usp.br (F.M.E.T.); luana.mendonca@usp.br (L.d.M.O.); pietrobonaj@usp.br (A.J.P.); alberto.duarte@hc.fm.usp.br (A.J.d.S.D.); 2 Department of Immunology, Institute of Biomedical Sciences, University of São Paulo, São Paulo 05508000, Brazil; erika.salles@gmail.com (É.M.d.S.); relima@usp.br (M.R.D.L.); 3 Department of Virology, Aggeu Magalhães Institute, Oswaldo Cruz Foundation, Recife 50740465, Brazil; isabelle.viana@fiocruz.br (I.F.T.V.); roberto.neto@fiocruz.br (R.D.L.); marques@pitt.edu (E.T.d.A.M.); 4 Laboratory of Immunobiology and Biomarkers, Center of Immunology, Institute Adolfo Lutz, São Paulo 01246000, Brazil; paula.rigato@ial.sp.gov.br; 5 Department of Infectious Diseases and Microbiology, University of Pittsburgh, Pittsburgh, PA 15213, USA

**Keywords:** DNA vaccine, neonate, HIV, immunogenicity, germinal center

## Abstract

Neonates have a limited adaptive response of plasma cells, germinal center (GC) B cells, and T follicular helper cells (T_FH_). As neonatal vaccination can be an important tool for AIDS prevention, these limitations need to be overcome. Chimeric DNA vaccine encoding p55Gag HIV-1 protein conjugated with lysosomal-associated membrane protein 1 (LAMP-1) has been described as immunogenic in the neonate period. Herein, we investigated the immunologic mechanisms involved in neonatal immunization with a LAMP-1/p55Gag (*LAMP/Gag*) DNA vaccine in a C57BL/6 mouse background. Neonatal *LAMP/Gag* vaccination induced strong Gag-specific T-cell response until adulthood and elevated levels of anti-Gag IgG antibodies. We also demonstrated for the first time that the immunogenicity of the neonatal period with *LAMP/Gag* is due to the induction of high-affinity anti-p24 IgG antibodies and long-term plasma cells. Together with that, there is the generation of early T_FH_ cells and the formation of GC sites with the upregulation of activation-induced cytidine deaminase (AID) enzyme mRNA and protein expression in draining lymph nodes after neonatal *LAMP/Gag* vaccination. These findings underscore that the LAMP-1 strategy in the chimeric vaccine could be useful to enhance antibody production even in the face of neonatal immaturity, and they contribute to the development of new vaccine approaches for other emerging pathogens at an early stage of life.

## 1. Introduction

HIV infection is an aggravating factor in public health worldwide. Currently, 38.4 million people are living with HIV [1]. A high number of exposed, uninfected children, as well as the vertical transmission of HIV infection, highlights the importance of developing anti-HIV strategies capable of generating a long-lasting immune response in neonates/children that covers maternal age.

Neonates have immune systems that are going through development and maturation processes, increasing their susceptibility to infections [2,3]. This neonatal immunological immaturity affects the immune tissues’ architecture and both arms of immunity, which are the innate and adaptive responses. Some of the peculiarities include the immaturity of lymphoid tissues, including the presence of immature follicular dendritic cells (FDCs), a reduction of T_FH_ cells, and a delay in the formation of GC sites, leading to functional impairment in antigen-specific antibody production [4,5]. In addition, the study of genetic vaccines and their evaluation of the dynamics of T_FH_ cells at the neonatal phase is underexplored.

Neonate vaccination could be affected by maternal antibodies [6], whereas neonatal DNA vaccination seems to overcome the inhibitory effect of maternal immunization, favoring neonatal antigen priming. At the site of injection, DNA vaccines delivered to cells mainly induce a CD8+ T-cell response [7]. A strategy linking antigen DNA to lysosomal-associated membrane protein 1 (LAMP-1) targets the antigen to MHC II molecules, successfully priming CD4+ T cells, and consequently CD8+ T cells, as well as antibody production by B cells [8,9]. To HIV, the chimeric DNA vaccine *LAMP/Gag* that associates the HIV-1 p55Gag protein with LAMP-1 induced robust CD4+ T-cell and CD8+ T-cell responses with strong and long-lasting humoral responses in adult mice [10,11,12] and a non-human primate model [13]. LAMP-1 arrives in specialized endosomal compartments called MIICs (endosomal MHC class II compartments), which direct the presentation of the antigen by MHC II molecules mainly to CD4+ T cells [8,9].

Our previous studies demonstrated that the *LAMP/Gag* DNA vaccine is immunogenic in BALB/c neonate mice, and it is also capable of increasing the cellular and humoral Gag-specific response, as well as a long-term response when compared to the vaccine that encodes the Gag protein alone [14]. Maternal *LAMP/Gag* DNA immunization interferes with the effect of *LAMP/Gag* in offspring, but it does not inhibit it; also, it is able to prime offspring in the uterus [15]. In addition, the *LAMP/Gag* DNA mucosal vaccination of neonatal mice is correlated with a strong cellular and humoral anti-Gag response [16]. We hypothesized that the chimeric vaccine LAMP-1 may overcome the GC immaturity by triggering T_FH_ cells, leading to antibody affinity maturation upon neonatal vaccination.

Some studies have evaluated the immunogenicity, at least in BALB/c (H-2d) and C57BL/6 (H-2b) mouse strains, each representing a distinct MHC haplotype [17]. In this context, the *LAMP/Gag* DNA vaccine response remains unknown in mouse strains other than BALB/c.

Therefore, we have shown that neonatal *LAMP/Gag* DNA vaccination in C57BL/6 mice induces the early activation of T_FH_ cells, as evidenced in draining lymph nodes within 3 days post-boost, as well as in GC sites, in this study. Here, we have presented, for the first time, a genetic vaccine strategy that improves the maturation of GCs with a high-affinity antibody production in neonatal immunization.

## 2. Material and Methods

### 2.1. Plasmids

Eukaryotic expression plasmid with a fragment of the HIV-1 HXB2 p55Gag gene, nucleotides 1–1503 (GenBank KO3455), was inserted into the pITR mammalian expression vector, which contains a cytomegalovirus promoter and adeno-associated virus-inverted terminal repeats flanking the expression elements. The *LAMP/Gag* chimera was made by inserting the p55Gag sequence between the luminal domain and the transmembrane/cytoplasmic tail of LAMP-1 (GenBank J03881) and cloned into the same vector [12]. All plasmids used for vaccination were produced as previously described [12,14].

### 2.2. Animals

C57BL/6 mice, aged 7 days or 8–10 weeks old, were purchased from the Biotério da Faculdade de Medicina da Universidade de São Paulo (FMUSP, São Paulo, Brazil) and bred in a specific pathogen-free animal facility. The protocol was approved by the Ethics Committee of Animal Care and Use (Comissão de Ética no Uso de Animais, permit CEUA-ICB: 9/2016 and CEUA-IMT: 333).

### 2.3. Mice Immunization Protocol

Adult mice (8–10 weeks of age) of both sexes were immunized twice by intradermal (ID) route at a 20-day interval with 50 μg of DNA vaccines encoding either *LAMP/Gag* or *Gag* plasmids. We immunized 7-day-old neonate mice of both sexes twice (ID) with 5 μg of the same DNA plasmids at a 25-day interval. Saline solution was used as a negative control. The analysis was performed at various times after the boost. For the long-lasting response, mice were analyzed 4 months after neonatal immunization; also, 7 days before the analysis, they received an additional dose (50 μg) of the respective vaccine.

### 2.4. Isolation of Mononuclear Cells

Spleens and inguinal lymph nodes (ILNs) were obtained aseptically, mashed through 40 µm cell-strainers (BD Bioscience, San Diego, CA, USA), centrifuged, and resuspended in an R-10 medium (RPMI 1640 medium with 10% fetal bovine serum—GIBCO, Carlsbad, CA, USA). Spleen mononuclear cells (MNCs) were isolated after centrifugation in Percoll 70% solution (GE Healthcare, Munich, Germany). After washing, the final cell pellet was diluted in an R10 medium. CD4+ or CD8+ T-cell-enriched populations were obtained by negative selection with magnetic columns (Miltenyi, San Diego, CA, USA) according to the manufacturer’s protocol. 

### 2.5. Detection of IFN-γ and Antibody-Secreting Cells by ELISPOT Assay

IFN-γ-producing cells from the spleen were assessed by ELISPOT assays, using an ELISPOT set from BD Biosciences (San Diego, CA, USA) [16]. MNCs (0.2—0.5 × 10^6^ cells/well) were stimulated with 1 µg/mL of anti-mouse CD3 and anti-CD28 antibodies (BD Bioscience, San Diego, CA, USA), 10 µg/mL of recombinant p24 HIV-1 (kindly provided by Prof. Luis Carlos de Souza Ferreira, Instituto de Ciências Biomédicas, Universidade de São Paulo, São Paulo, Brazil) or 10 μg/mL of 12 pools of HIV-Gag peptides (15-mers of the HIV-1 HXB2 Gag peptide, with overlaps of 11 residues; NIH AIDS Research and Reference Reagent Program). Spots were quantified with an AID *i*Spot FluoroSpot Reader System using the software AID EliSpot version 7.0 (AID—Autoimmun Diagnostika GMBH, Strassberg, Germany). The reaction was considered positive when the number of spot-forming cells (SFCs) was equal to or higher than 10 SFC/10^6^ cells. All results are expressed as the mean number of SFCs per 10^6^ cells. Antigen-specific plasma cells were determined by B-cell ELISPOT assay, using 5 µg/mL of p24 HIV-1 for coating, followed by MNCs (1 × 10^6^ cells/well) and biotinylated anti-IgG1 (Southern Biotech, Birmingham, AL, USA). This reaction was revealed using streptavidin-HRP and AEC substrate (BD Bioscience, San Diego, CA, USA), according to the manufacturer’s protocol. The results were converted to Ab-secreting cells per million cultured cells (ASC/10^6^ cells).

### 2.6. Detection of Anti-P24 IgG Antibodies through ELISA

Mouse serum was obtained at different time points after the immunizations occurred, and anti-Gag antibody levels were measured by ELISA. High-binding, half-area 96-well microplates (Costar, Washington, DC, USA) were coated with 1 µg/mL of recombinant p24 HIV-1 for 16 h at 4 °C. The reactions were blocked with 5% skim milk (Bio-Rad, Hercules, CA, USA) in PBS-T buffer (1× PBS with 0.05% Tween-20) for 15 min at room temperature (RT). Next, samples were added and diluted at 1:50 in an assay buffer (5% skim milk in PBS-T) for 2 h at RT. Plates were washed 5 times with PBS-T and incubated with peroxidase-conjugated anti-mouse IgG antibody (Sigma Aldrich, St. Louis, MO, USA) for 1 h at RT. After washing the set, the reaction was revealed with tetramethylbenzidine TMB-KPL substrate (TMB, KPL SureBlue Reserve^TM^) for 30 min at RT, stopped by 1N HCl, and read at 450 nm (OD450 nm) using a microplate spectrophotometer Benchmark Plus (Bio-rad, Hercules, CA, USA). All samples were tested in duplicate, and the intra-assay variability was below 20%.

### 2.7. T_FH_ Cells and GC B Cell Characterization by Flow Cytometry

The frequency of T_FH_ and GC B cells from draining ILNs was assessed by flow cytometry. For T_FH_, 1 × 10^6^ cells were stained with anti-CD3 (PerCP-Cy5.5), anti-CD4 (V500), anti-CD8 (APC-Cy7), anti-CD19 (Alexa 700), anti-CXCR5 (biotin), streptavidin (APC), anti-PD-1 (PE), and anti-Bcl-6 (V450) (BD Biosciences, San Diego, CA, USA). Intracellular staining for Bcl-6 was performed with a Foxp3 staining kit (eBioscience, Carlsbad, CA, USA) according to the manufacturer’s instructions. Data was expressed as the frequency (%) of CD4+ T cells expressing CXCR5 + PD-1 + BCL-6+ markers. For GC B cells, 1 × 10^6^ cells were stained with anti-CD3 (PerCP-Cy5.5), anti-CD4 (V500), anti-CD19 (Alexa 700), anti-CD95 (PE-Cy7), and anti-GL-7 (FITC) (BD Biosciences). Data was expressed as the frequency (%) of B cells expressing CD95 + GL-7+ markers. A total of 200,000 events were collected and analyzed by flow cytometry (LSR Fortessa, BD Biosciences, San Diego, CA, USA) using the FACS Diva (BD Bioscience, San Diego, CA, USA) and FlowJo 10.0.6 (BD Bioscience, San Diego, CA, USA) software programs. Fluorescence Minus One (FMO) controls were performed to confirm proper compensation and define positive signals.

### 2.8. Immunofluorescence

Slices with a 5 μm thickness were fixed in acetone and blocked with 1% BSA plus Fc Block (1:100) (BD Bioscience, San Diego, CA, USA) for 30 min at 25 °C. Next, the slides were stained with anti-CD4 (PE) and anti-GL-7 (FITC) (BD Biosciences, San Diego, CA, USA) or anti-CD19 (PE) (BD Biosciences, San Diego, CA, USA) and primary anti-AID antibodies (Abcam, Waltham, MA, USA) for 2 h at 25 °C. For AID, secondary anti-rabbit FITC antibodies (Abcam, Waltham, MA, USA) were added for 1 h at 25 °C. After washing, slides were mounted with Anti-Fade Fluorescence Mounting Medium (Abcam, Waltham, MA, USA). Sections were analyzed by immunofluorescence microscopy (AxioVert.A1/Zeiss, Jena, Germany).

### 2.9. Real-Time PCR

Total RNA was extracted from ILNs cells using an RNeasyPlus Mini Kit (Qiagen, Valencia, CA, USA), followed by reverse transcription using the Reverse Transcriptase Kit (Qiagen, Valencia, CA, USA). Primers used for real-time PCR assays were: AID—F: GCCACCTTCGCAACAAGTCT/R: CCGGGCACAGTCATAGCAC; Bcl-6: F: CACACCCGTCCATCATTGAA/R: TGTCCTCACGGTGCCTTTTT; BLIMP-1: F: GGCTCCACTACCCTTATCCTG/R: TCCTTTTGGAGGGATTGGAGTC; IL-21—F: TGAAAGCCTGTGGAAGTGCAAACC/R: AGCAGATTCATCACAGGACACCCA; and GAPDH: F: TTCACCACCATGGAGAAGGC/R: GGCATGGACTGTGGTCATGA.

GAPDH levels were used to normalize the mRNA. Real-time quantitative PCR was performed using an automated sequencer (Model 7500; Applied Biosystems, Carlsbad, CA, USA) with specific primers and SYBR Green (Applied Biosystems, Carlsbad, CA, USA). The amplification results were analyzed using Sequence Detection System (SDS) software (Applied Biosystems, Carlsbad, CA, USA), and a normalized expression was calculated [18].

### 2.10. Anti-P24 Antibody Affinity by Microscale Thermophoresis

The affinity of the anti-Gag IgG antibodies was quantified using a microscale thermophoresis assay (MST), as previously described [19]. The p24 HIV protein (20 μM) was labeled using a RED fluorescent dye NT-647-NHS Labeling Kit (NanoTemper Technologies, Munich, Germany), according to the manufacturer’s instructions. Unreacted dye was removed by buffer-exchange chromatography (EC), while labeled proteins were eluted in 1× PBS buffer and quantified through spectrophotometry (Nanodrop One^c^, ThermoFisher Scientific, Waltham, MA, USA). The total protein content was also measured using spectrophotometry for all serum samples used. Binding assays between the HIV-1 p24 protein and anti-Gag antibodies in the mouse serum were conducted with 150 nM of labeled p24 protein and a series of 16 2-fold serial dilutions of 2 representative mouse sera in 1× PBS, 0.05% Tween-20 buffer (starting from 114 µM and 46.7 µM for animals 3 and 6, respectively). Measurements were performed in MST-standard-coated capillaries at 20 °C using a Monolith NT.115 (NanoTemper Technologies, Munich, Germany). The red excitation LED was set to 40% and the laser power to medium. The laser on-time was set to 30 s, and the laser off-time was set to 5 s. The measured affinity was considered as an apparent affinity (defined here as half-maximum binding parameter (EC50), given the lack of a 1:1 ratio IgG-p24 protein, since total serum polyclonal IgG was used in the assay. The EC50 was derived from 3 independent experiments. The quality of each MST run was assessed by performing a capillary scan before and after each run to check that the fluorescence between samples remained within a 10% variation.

### 2.11. Statistical Analysis

Data were analyzed using GraphPad Prism software (Version 6.0). Comparisons between groups were performed using the non-parametric Mann–Whitney test. The level of significance was considered when *p* ≤ 0.05.

## 3. Results

### 3.1. Neonatal Chimeric LAMP/Gag DNA Vaccine Enhances T-Cell Response

Considering the strong CD4+ T-cell activation and antibody production in the neonatal BALB/c *LAMP/Gag* DNA-immunized mice [14], we aimed to verify the mechanism involved in these vaccine responses using C57BL/6 mice. First, we evaluated the immunogenicity of the chimeric *LAMP/Gag* DNA vaccine in C57BL/6 mice, accessing the profile of T-cell response to all of the HIV-1 Gag peptides. No adverse reaction or death were observed in the immunized animals in either the adult or the neonatal stage.

We observed in the C57BL/6 mice class II immunodominant peptide-recognizing pools, located between 121–171aa and 281–331aa (Supplementary Figure S1A), similar to that in the BALB/c mice, whose sequences were between 241–291aa and 281–331aa [10]. However, for MHC class I, the more-recognizable peptide sequence was between 361–411aa (Supplementary Figure S1B), different from the BALB/c sequence 181–227aa, which includes the immunodominant-class-I-restricted epitope AMQMLKETI (197–205aa) [20]. We showed that, for the C57BL/6 (H-2b) haplotype, along the 361–411aa sequence, the CD8 response was restricted to the peptides corresponding to sequences 361–375aa (Supplementary Figure S1C).

**Figure 1:**
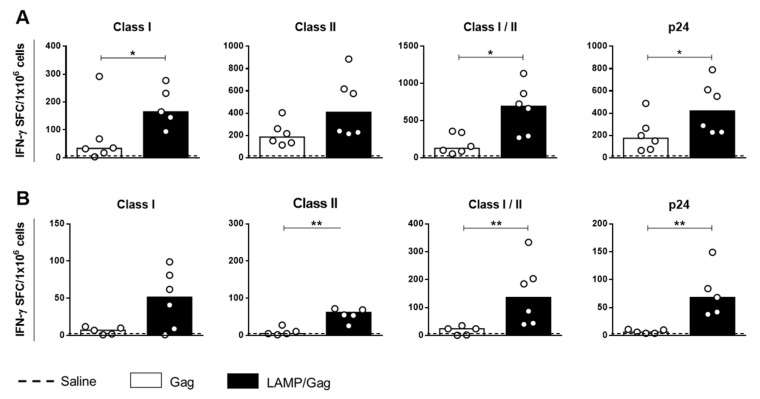
Generation of Gag-specific cellular response by neonatal C57BL/6 mouse immunization. Neonate C57BL/6 mice were immunized (ID) with 2 doses of *LAMP/Gag* or *Gag* DNA vaccines at a 25-day interval: (**A**) Gag-specific IFN-γ-secreting cells were evaluated 10 days after boost by ELISPOT assay in response to class I (361–411aa), class II (121–171aa and 281–331aa), and class I plus II immunodominant peptide pools or p24 recombinant protein; (**B**) The cellular response was evaluated, as shown in (**A**) 4 months after immunization. Saline was used as a negative control (*n* = 6). Assays were performed in duplicate, and the results were subtracted from the baseline value. The data are shown as medians. * *p* ≤ 0.05; ** *p* ≤ 0.01.

Figure 1A shows that neonatal *LAMP/Gag* DNA immunization increased IFN-γ-secreting cells in comparison to the *Gag* vaccine to the MHC-class-I-restricted peptides; promiscuous peptides recognized by both MHC class I/II molecules and p24 recombinant protein. In addition, the long-lasting response at 4 months after neonatal *LAMP/Gag* immunization showed a decreased frequency of IFN-γ-secreting cells over time, whereas it maintained an anti-Gag cellular response to the class II peptides, not only those of class I, as well as to mixed-class I/II peptides and p24 protein, as compared to the native *Gag* vaccine (Figure 1B). Curiously, this profile was similar to the one observed in adult immunization (Supplementary Figure S2A), indicating that vaccination during the early phase generates antigen-specific T cells which remain into adulthood.

### 3.2. LAMP/Gag Vaccination of Neonates Generates High-Affinity Antibodies and Long-Lived Plasma Cells 

**Figure 2:**
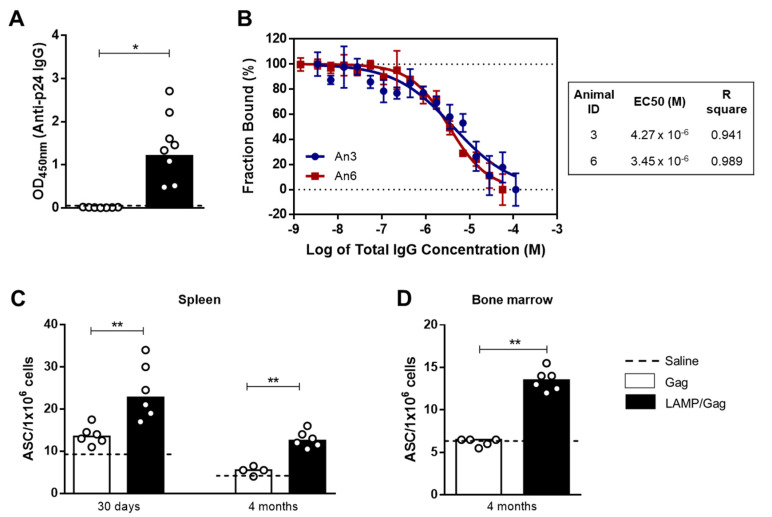
Gag-specific humoral response and plasma-cell generation. Neonate mice were immunized with *Gag* or *LAMP/Gag* vaccines: (**A**) Anti-p24 (HIV-1) IgG antibodies were detected by ELISA in the serum obtained from immunized mice within 10 days after the boost (Saline group: *n* = 4); (**B**) Anti-Gag IgG antibody binding curves to the HIV-1 p24 protein were measured by microscale thermophoresis, where serum binding curves acquired from 2 selected mouse-serum samples were plotted in terms of fraction bound (%) vs. concentration of total IgG; (**C**) The number of anti-p24 IgG1 ASCs was assessed in the spleen by ELISPOT for B cells in the short-term (30 days) and long-term (4 months) after immunization (Saline group: *n* = 3–6); (**D**) The number of ASCs was also evaluated in the bone marrow at 4 months. The data are shown as medians. * *p* ≤ 0.05; ** *p* ≤ 0.01.

Neonatal *LAMP/Gag* immunization was also effective in generating a humoral response in C57BL/6 mice in comparison to *Gag* alone (Figure 2A). Immunogenicity was also confirmed in adult *LAMP/Gag* immunization, which induced elevated levels of anti-p24 IgG antibody (Supplementary Figure S2B,C).

Gag antigen is not able to induce neutralizing antibodies (nAb) generation, which are developed in response to viral surface antigens. Therefore, here we evaluated the antibodies’ affinity as a complementary, functional mechanism of the vaccine humoral response. Antibody-binding affinity between antigen-specific antibodies in the sera from 2 randomly selected *LAMP/Gag* immunized neonates and the p24 protein was also measured through microscale thermophoresis. As expected, anti-p24 IgG antibodies were observed to bind with high apparent affinity to the p24 protein (since total serum polyclonal IgG were included in the experiment). The half-maximum binding parameter (EC50) between IgG antibodies and p24 was determined as being 4.27 × 10^−6^ M and 3.45 × 10^−6^ M for animals 3 and 6, respectively (Figure 2B).

It is known that neonatal *LAMP/Gag* immunization is capable of generating antibodies for long periods [14]. However, the evaluation of plasma cells has not yet been described. Next, we assessed whether the chimeric *LAMP/Gag* DNA vaccine could increase the frequency of antigen-specific plasma cells in the spleen and bone marrow. For this, we evaluated the number of anti-p24 IgG1 antibody-secreting cells (ASCs) by B-cell ELISPOT assay. The frequency of IgG1 anti-Gag plasma cells was increased in *LAMP/Gag* as compared to *Gag* immunization over short and extended periods after vaccine boost in the spleen (Figure 2C). Similar findings were observed 4 months after the immunization in the bone marrow (Figure 2D). It is worth mentioning that, for the long-lasting protocol, the mice received an additional dose 7 days before the analysis in order to boost the response, which possibly favored the targeting of plasma cells to the periphery.

Therefore, these results show that neonatal *LAMP/Gag* immunization promotes a generation of antigen-specific long-lived plasma cells. 

### 3.3. Neonatal LAMP/Gag Immunization Promotes Early T_FH_ Cell Generation

**Figure 3:**
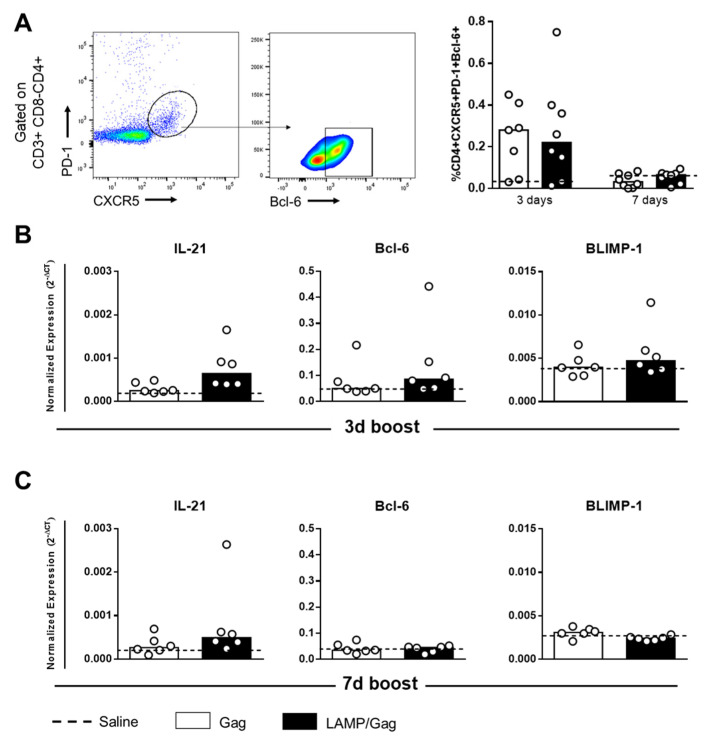
T_FH_ cell generation in draining inguinal lymph nodes. Neonate mice were immunized with *LAMP/Gag* or *Gag* DNA vaccines, and the analysis was performed in ILNs within 3 or 7 days after the boost: (**A**) Frequency of T_FH_ cells (CD4 + CXCR5 + PD-1 + Bcl6 +) in ILNs by flow cytometry (Saline group: *n* = 5); (**B**,**C**) IL-21, Bcl-6, and BLIMP-1 mRNA expression evaluated in ILNs by real-time PCR at 3 and 7 days after boost, respectively.(Saline group: *n* = 3–6). The data are shown as medians.

Our results show that only the *LAMP/Gag* DNA vaccine was able to generate a robust humoral response. To better understand the mechanism involved, we evaluated the frequency of T_FH_ cells in draining ILNs by neonatal immunization with our DNA vaccines at two different time points during the first-week post-boost. T_FH_ cells were characterized according to CD3 + CD4 + CXCR5 + PD-1 + Bcl-6+ phenotype (Supplementary Figure S3A). Similar frequencies of T_FH_ cells were detected between the groups immunized with the *Gag* or *LAMP/Gag* DNA vaccines (Figure 3A). Despite the early generation of T_FH_ cells that followed immunization within 3 days after the boost, these cells were not increased within 7 days after the boost.

T_FH_ cell polarization is dependent on the action of IL-21 cytokine and the Bcl-6 transcription factor, which is regulated by BLIMP-1 [21]. Evidence of these factors in mRNA expression was barely detected in groups submitted to neonatal immunization during the first week after the boost (Figure 3B). It seems that the transcripts appeared in an early period.

Although the T_FH_ cell frequency has been similar, the functional capacity to induce an adequate B-cell response is quite different between *Gag* and *LAMP/Gag* immunization.

### 3.4. LAMP/Gag Induces Germinal Center Sites with Up-Regulation of AID Enzyme Expression

Considering that neonatal immunization led to T_FH_ cells just 3 days after the boost, to better understand the functional aspects involved, we checked GC markers at the draining lymph nodes at this time point.

**Figure 4:**
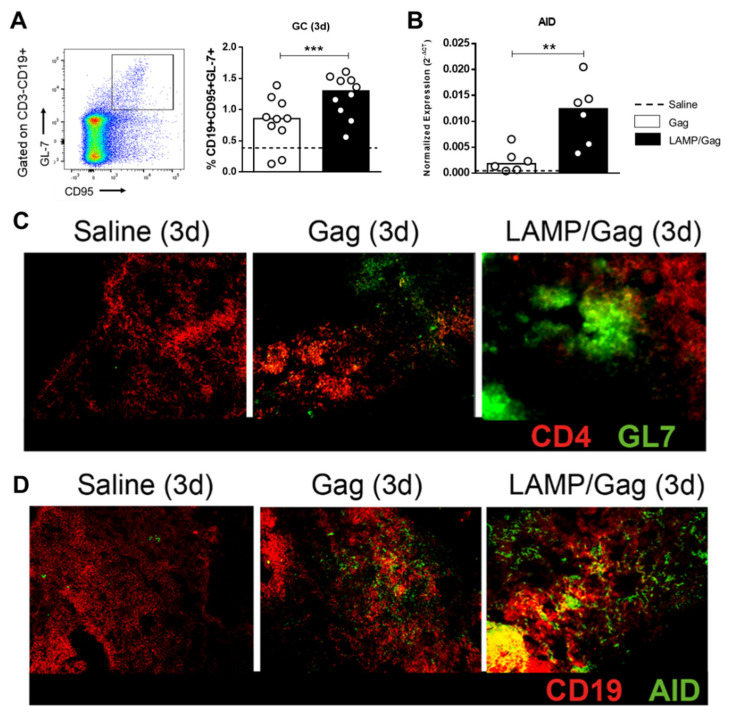
GC site formation and antibody-binding affinity: (**A**) The frequency of GC B cells (CD19 + CD95 + GL-7+) was evaluated within 3 days after the boost in neonatal immunization in ILNs by flow cytometry (Saline group: *n* = 8); (**B**) mRNA expression of genes for AID enzyme by real-time PCR of cells obtained from ILNs 3 days post-boost (Saline group: *n* = 3); (**C**) Representative ILN sections from immunized neonates (*n* = 3/group) were obtained within 3 days after the boost, and GC was identified by immunofluorescence (CD3: red; GL-7: green; original magnification ×10); (**D**) Representative expression of AID in ILN tissues by immunofluorescence as described in B (CD19: red; AID: green; original magnification ×10). ** *p* ≤ 0,01; *** *p* ≤ 0001.

First, we observed that the *LAMP/Gag* DNA vaccine increased the frequency of GC B cells in the ILNs compared to native *Gag*, according to the CD3-CD19 + CD95 + GL-7+ phenotype (Figure 4A; Supplementary Figure S3B). 

In addition to the help of T_FH_ cells, B-cell activity is dependent on the action of the AID enzyme, which mediates class-switch recombination and somatic hypermutation, leading to antibody affinity maturation. Interestingly, neonatal immunization with *LAMP/Gag* was able to up-regulate AID mRNA expression compared to the native *Gag* vaccine (Figure 4B). 

The formation of GC sites was confirmed by immunofluorescence in ILN tissue sections, in which the *LAMP/Gag* DNA vaccine induced GCs that were more evident and had a better structural organization than did the *Gag* (Figure 4C). Also, we observed an increased AID protein expression in B cells, mainly following *LAMP/Gag* vaccination (Figure 4D).

Taken together, our results provide evidence that the *LAMP/Gag* vaccine strategy is functional during this period, being crucial to promoting the maturation of the humoral response.

## 4. Discussion

Considering that only the *LAMP/Gag* DNA vaccine is able to be immunogenic, activating CD4+ T cells and inducing strong anti-Gag antibodies in neonatal immunization, we evaluated the mechanisms involved in the enhancement of the humoral response. Our results showed that the LAMP-1 chimeric to p55Gag antigen strategy leads to early GC response, up-regulating AID expression, and favoring high-affinity antibody generation.

In response to *LAMP/Gag* immunization, we have C57BL/6 mice recognizing the class I immunodominant peptides from the Gag region of HIV, different from BALB/c mice, despite both mouse strains recognizing similar class II immunodominant peptides. The class I immunodominant sequence (aa361–411) corresponds to the p15 protein [22,23], and class II sequences (aa121–171 and aa281–331) are related to the p24 protein portion. In BALB/c mice, both CD4 and CD8 T-cell responses were directed to the portion referring to p24 [10]. It has been described that the cellular response’s breadth and immunodominance can vary between different strains of mice in vaccine models for HIV [24,25]. Consequently, some variation in class I immunodominance in C57BL/6 mice immunized with *LAMP/Gag* was expected. 

Studies with HIV-infected individuals have shown the presence of a specific cytotoxic T-cell response to p15 Gag and suggest this as a good prognosis [26,27,28]. In comparison to *Gag* alone, neonatal *LAMP/Gag* immunization increased IFN-γ-secreting cells for the class I immunodominant, which could reflect the role of p15 protein on the generation of cytotoxic response by immunization. Despite the reduction in the frequency, 4 months after the immunization, *LAMP/Gag* maintained an increase in the cellular response specifically to immunodominants related to the p24 region, suggesting that a specific T-helper response remains until adulthood. This profile was similar to that observed in adult immunization.

As Gag is an internal viral protein, anti-Gag antibodies do not have a neutralizing capacity. Once the cells are infected, the neutralizing action is not the only humoral mechanism. IgG1 anti-p24 antibodies were associated with Fc receptor (FcγR)-mediated viral control in HIV-infected individuals, leading to antibody-dependent phagocytosis and antibody-dependent cellular cytotoxicity (ADCC) [29]. In addition, *LAMP/Gag* immunization induced high-affinity antibodies. Therefore, anti-Gag antibodies induced by the *LAMP/Gag* vaccine may contribute to the ADCC-mediated removal of HIV-infected cells.

Here, we show for the first time that neonatal *LAMP/Gag* immunization is able to increase the frequency of long-term anti-Gag IgG1-secreting cells in the spleen and bone marrow, evidencing the capacity of this vaccine to develop long-lived plasma cells. This may be the result of adequate T_FH_ cell activation and GC reactions.

Our data show an early and transitory presence of T_FH_ cells (CD4 + CXCR5 + PD-1 + Bcl-6+) in the draining ILNs within 3 days after boosting neonatal immunization, similar to the *Gag* and *LAMP/Gag* DNA vaccine groups. These cells decrease over time, and it is well-established that the expansion and differentiation of T_FH_ cells are limited in the neonatal phase, which are mainly located in the interfollicular regions [30,31]. T_FH_ cells help the B-cell response in follicles on lymphoid secondary tissues, where they are essential for the formation of GC sites, being crucial in the development of memory B cells and long-lived plasma cells [32]. The presence of T_FH_ cells was associated with the magnitude of the anti-SIV humoral response in the lymph nodes of non-human primates [33].

DNA vaccination studies in neonatal mouse models have induced great immunogenicity, protective immunity, and long-lived responses [34,35,36]. However, there is no description of T_FH_ cell activity or other related mechanisms in these studies. T_FH_ cell dynamics in early-life immunization have been built mainly on protein immunization models [5,30,31] rather than on genetic approaches, which could assure a less-robust activation.

Although there is no difference in frequency between groups, the effect or response seems to be different. In addition, we have to consider that neonatal cells have high plasticity [2,37]. In this sense, our findings show that the degree of T-helper-cells activation via LAMP-1 could favor the formation of GCs in the neonatal period through an increase in the recognition of the epitope by CD4 T cells, which contributes to the vaccine response’s maturation. Additionally, changes in the interaction between T_FH_ cells and B cells, mediated by targeting via LAMP, could be another mechanism involved. In this way, future functional cell assays may contribute to the knowledge of new pediatric vaccine approaches. In fact, neonatal *LAMP/Gag* immunization increased the frequency of GC B cells as compared to *Gag* alone and also promoted the early formation of GC sites in draining lymph nodes with a better structural organization than only the *Gag* vaccine.

T_FH_-cell activation leads to GC reactions, characterized by somatic hypermutation with class-switch antibody and affinity maturation; also, this phenomenon is dependent on the activity of the AID enzyme in activated B cells [38,39]. During the neonatal phase, the architecture of the secondary lymphoid organs is not adequately formed to promote the full antibody response in the first weeks of life [40]. Here, we checked some factors involved in the function of T_FH_ and B cells. In the cells obtained from the ILNs during the first week post-boost, the mRNA expressions of the IL-21, Bcl-6, and BLIMP-1 genes were similar between the vaccinated groups, possibly due to the expression kinetics of each of these genes. However, neonatal *LAMP/Gag* immunization promoted the up-regulation of the AID enzyme at the mRNA- and protein-expression levels, compared to *Gag,* within 3 days after the boost. This was confirmed by the high-affinity antibodies in *LAMP/Gag* DNA immunized mice.

The neonatally immunized mice with a protein (DNP-KLH) showed an accelerated structural maturation of lymph nodes, but not in the response of the B cells, which occurred still later, and with reduced ability in the expression of AID, compromising the IgG response at this stage [41]. Nevertheless, there is no evidence of AID expression in models of DNA neonatal immunization.

Some studies have indicated that the use of Toll-like-receptor agonists and other adjuvants in neonatal vaccination are able to collaborate for a better T_FH_ response and, consequently, improve the induction of GCs [42,43,44,45,46]. In our model, we did not use any type of adjuvant formulation associated with the vaccine; therefore, the action of LAMP in contributing to an increase in the vaccine humoral response is evident as a kind of molecular adjuvant.

The development of a prophylactic HIV vaccine remains a challenge. Several candidate vaccines have not been successful in clinical trials [47,48,49,50,51]. At the moment, the only successful HIV clinical trial is the RV144 study (with an efficacy around 31%), in which the efficacy was correlated with an immunodominant epitope in the variable 2 (V2) domain of the HIV-1 envelope glycoprotein [52,53]. In addition, a recent study has proposed the rational design of combinatorial vaccines covering the V2 region as a potential strategy for improving vaccine efficacy [54]. Considering the viral-envelope protein heterogeneity, targeting strategies to more conserved antigens, such as the Gag protein, may be attractive [55]. In this way, LAMP-based, multi-epitope DNA vaccines targeted to the V2 region and Gag protein may represent a promising approach in inducing anti-HIV neutralizing antibodies, as well as a robust cellular response.

Taken together, the results confirm the chimeric DNA *LAMP-1/p55Gag* vaccine immunogenicity in C57BL/6 mice in the neonatal period, which contributes to the early development of T_FH_ cells capable of increasing the GC B cells. Interestingly, an adequate AID activation during this phase of life could be one of the mechanisms that makes *LAMP/Gag* effective in the enhancement of antibody production, even in the face of neonatal immaturity. The LAMP-1 strategy in the chimeric vaccine promotes intracellular antigen targeting of the CD4 T cells and the generation of essential effector responses. Moreover, understanding vaccine models capable of overcoming immunological immaturity at an early stage of life contributes to the development of innovative approaches, not only for HIV, but also for other emerging pathogens.

## Data Availability

Data are included in the article and are also available on request from the corresponding author.

## Supplementary Materials

The following are available online at https://www.mdpi.com/article/10.3390/vaccines10081246/s1, Figure S1. Characterization of Gag-immunodominant peptides in C57BL/6 (H-2b) mice, Figure S2. Immunogenicity of LAMP/Gag vaccine at adult immunization, Figure S3. Representative TFH cells and GC B cells strategy analysis.
