# Supplementary material for: LAMP-1 Chimeric to HIV-1 p55Gag in the Immunization of Neonate Mice Induces an Early Germinal Center Formation and AID Expression
Source: Vaccines (Basel). 2022 Aug 3;10(8):1246. doi: 10.3390/vaccines10081246 (PMC9414238; doi:10.3390/vaccines10081246)
Supplement: Supplementary file 1 [file vaccines-10-01246-s001.zip › vaccines-1820081-supplementary.pdf]

Supplementary Material

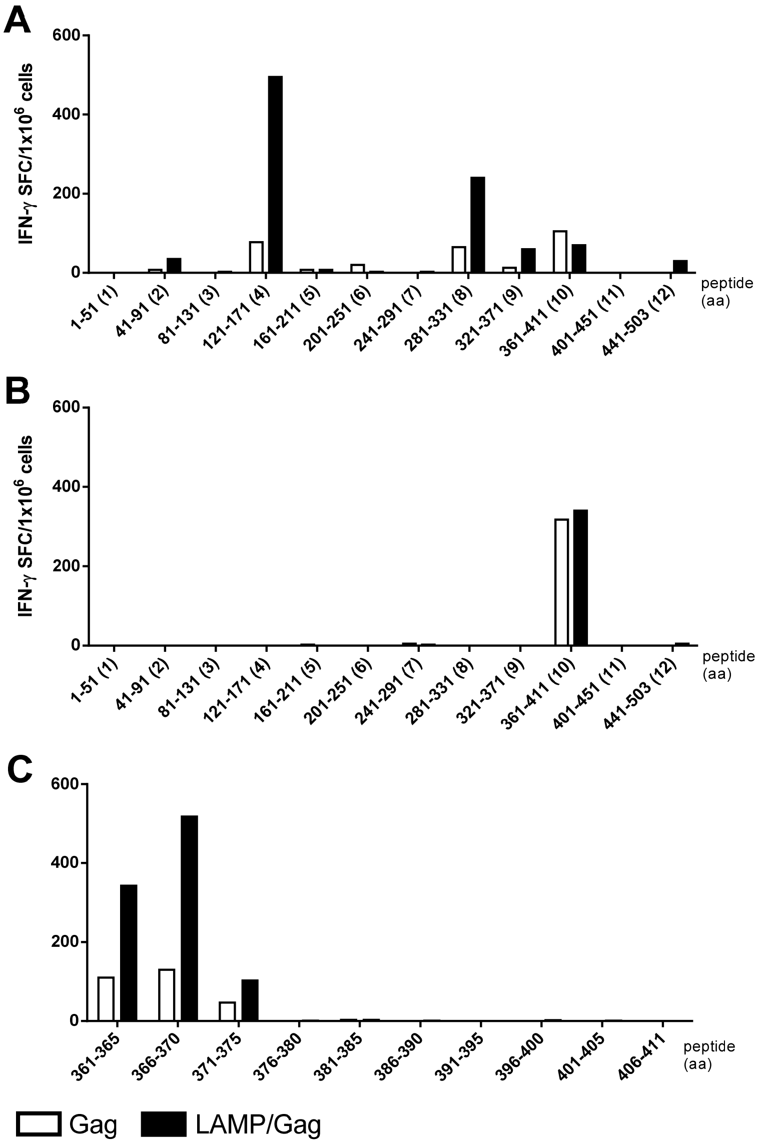

Figure S1. Characterization of Gag-immunodominant peptides in C57BL/6 (H-2^b) mice. Adult C57BL/6 mice were immunized (i.d.) with two doses of *LAMP/Gag* or Gag DNA vaccine at a 20-days interval and assessed by ELISPOT for IFN-γ with 12 pools of HIV-1 Gag protein peptides, using populations depleted of CD8+ T cells (A) or depleted of CD4+ T cells (B). (C) Peptides corresponding to pool 10 (aa361-411) were evaluated individually to identify the sequences restricted to MHC class I, using total MNCs. Representative of three independent experiments. The tests were performed in duplicate, and the results are expressed and subtracted from the baseline value.

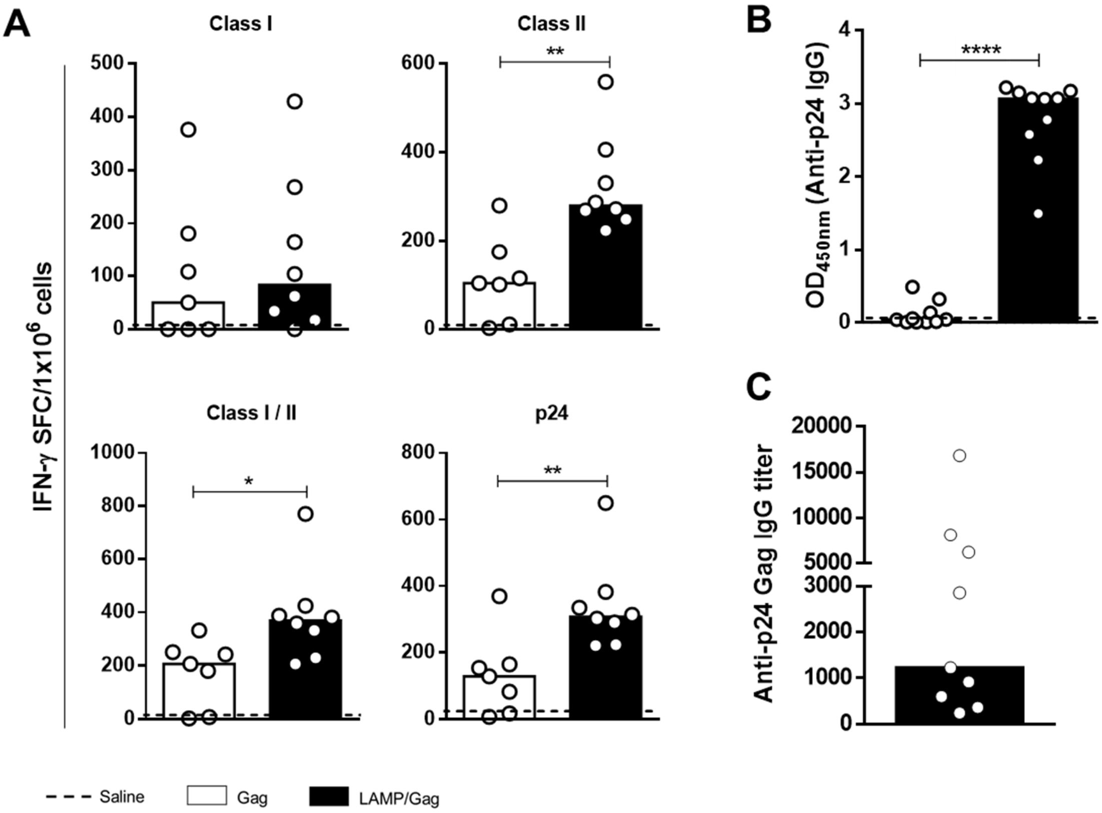

Figure S2. Immunogenicity of *LAMP/Gag* vaccine at adult immunization. Adult C57BL/6 mice were immunized (i.d.) with two doses of *LAMP/Gag* or *Gag* vaccines and the analysis was performed ten days after the boost. (A) Splenocytes were assessed by ELISPOT for IFN-γ in response to immunodominant Gag peptides or p24 protein. (B) Anti-p24 (HIV-1) IgG antibodies were detected by ELISA in the serum obtained from mice immunized with Gag or *LAMP/Gag* vaccines within ten days after the boost. (C) Anti-Gag IgG titration in serum from *LAMP/Gag* mice. Saline was used as a negative control. The data are plotted as median. * $p \leq 0{,}05$; ** $p \leq 0{,}01$; ** $p \leq 0{,}0001$.

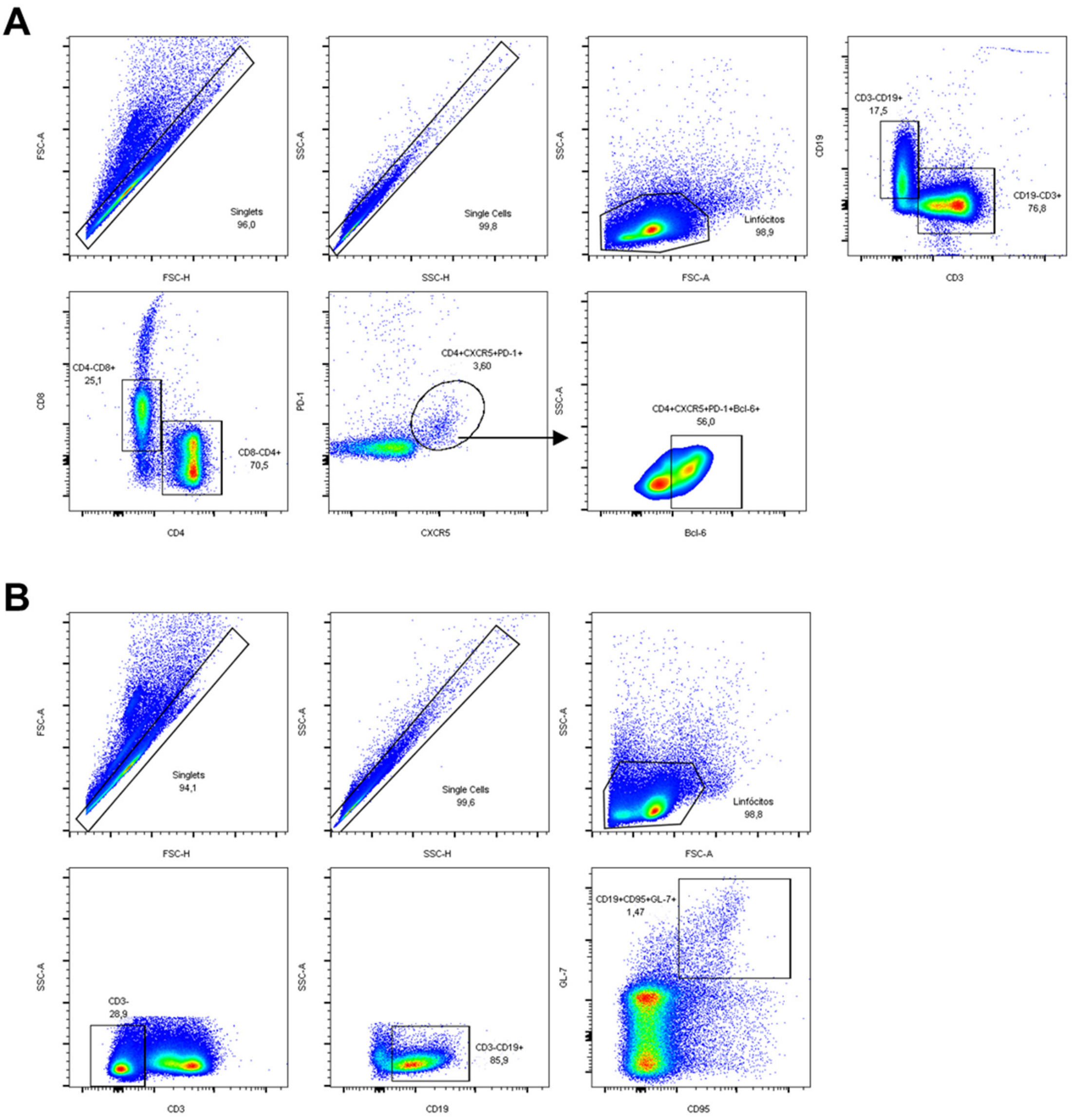

Figure S3. Representative T_{FH} cells and GC B cells strategy analysis. iLNs cells obtained at 3 days after boost from *LAMP/Gag* neonatal immunization were assessed by flow cytometry according to gate strategy to (A) T_{FH} cells phenotype CD3+CD4+CXCR5+PD-1+BCL-6+ and (B) germinal center B cells phenotype CD3-CD19+CD95+GL-7+.
